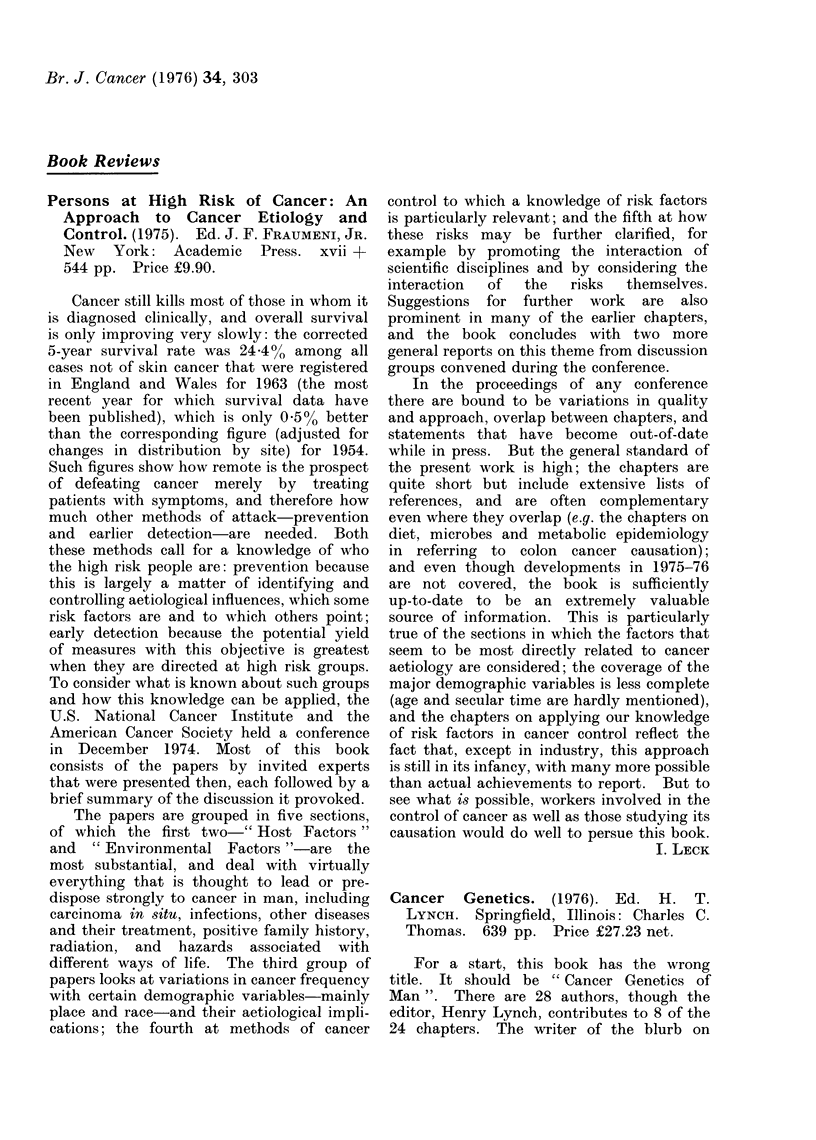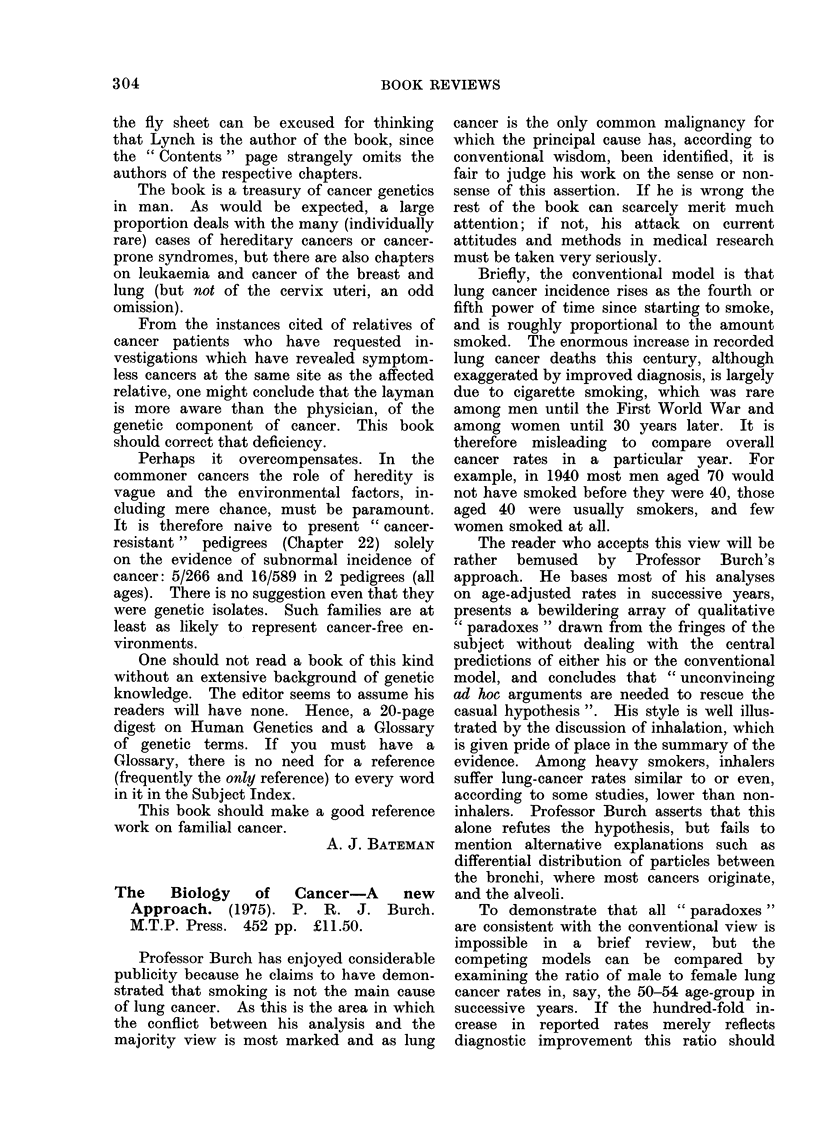# Cancer Genetics

**Published:** 1976-09

**Authors:** A. J. Bateman


**Cancer Genetics.** (1976). Ed. H. T. Lynch. Springfield, Illinois: Charles C. Thomas. 639 pp. Price £27.23 net.

For a start, this book has the wrong title. It should be " Cancer Genetics of Man ". There are 28 authors, though the editor, Henry Lynch, contributes to 8 of the 24 chapters. The writer of the blurb on the fly sheet can be excused for thinking that Lynch is the author of the book, since the " Contents " page strangely omits the authors of the respective chapters.

The book is a treasury of cancer genetics in man. As would be expected, a large proportion deals with the many (individually rare) cases of hereditary cancers or cancer-prone syndromes, but there are also chapters on leukaemia and cancer of the breast and lung (but *not* of the cervix uteri, an odd omission).

From the instances cited of relatives of cancer patients who have requested investigations which have revealed symptomless cancers at the same site as the affected relative, one might conclude that the layman is more aware than the physician, of the genetic component of cancer. This book should correct that deficiency.

Perhaps it overcompensates. In the commoner cancers the role of heredity is vague and the environmental factors, including mere chance, must be paramount. It is therefore naive to present " cancer-resistant " pedigrees (Chapter 22) solely on the evidence of subnormal incidence of cancer: 5/266 and 16/589 in 2 pedigrees (all ages). There is no suggestion even that they were genetic isolates. Such families are at least as likely to represent cancer-free environments.

One should not read a book of this kind without an extensive background of genetic knowledge. The editor seems to assume his readers will have none. Hence, a 20-page digest on Human Genetics and a Glossary of genetic terms. If you must have a Glossary, there is no need for a reference (frequently the *only* reference) to every word in it in the Subject Index.

This book should make a good reference work on familial cancer.

A. J. BATEMAN